# Influenza Surveillance Among Children With Pneumonia Admitted to a District Hospital in Coastal Kenya, 2007–2010

**DOI:** 10.1093/infdis/jis536

**Published:** 2012-12-15

**Authors:** Clayton O. Onyango, Regina Njeru, Sidi Kazungu, Rachel Achilla, Wallace Bulimo, Stephen R. Welch, Patricia A. Cane, Rory N. Gunson, Laura L. Hammitt, J. Anthony G. Scott, James A. Berkley, D. James Nokes

**Affiliations:** 1 KEMRI–Wellcome Trust Research Programme, Kilifi; 2 National Influenza Centre, Nairobi, Kenya; 3 Health Protection Agency, London; 4 Nuffield Department of Clinical Medicine, University of Oxford, Oxford; 5 School of Life Sciences, University of Warwick, Coventry, United Kingdom; 6 West of Scotland Specialist Virology Centre, Gartnavel General Hospital, Glasgow, Scotland

## Abstract

**Background:**

Influenza data gaps in sub-Saharan Africa include incidence, case fatality, seasonal patterns, and associations with prevalent disorders.

**Methods:**

Nasopharyngeal samples from children aged <12 years who were admitted to Kilifi District Hospital during 2007–2010 with severe or very severe pneumonia and resided in the local demographic surveillance system were screened for influenza A, B, and C viruses by molecular methods. Outpatient children provided comparative data.

**Results:**

Of 2002 admissions, influenza A virus infection was diagnosed in 3.5% (71), influenza B virus infection, in 0.9% (19); and influenza C virus infection, in 0.8% (11 of 1404 tested). Four patients with influenza died. Among outpatients, 13 of 331 (3.9%) with acute respiratory infection and 1 of 196 without acute respiratory infection were influenza positive. The annual incidence of severe or very severe pneumonia, of influenza (any type), and of influenza A, was 1321, 60, and 43 cases per 100 000 <5 years of age, respectively. Peak occurrence was in quarters 3–4 each year, and approximately 50% of cases involved infants: temporal association with bacteremia was absent. Hypoxia was more frequent among pneumonia cases involving influenza (odds ratio, 1.78; 95% confidence interval, 1.04–1.96). Influenza A virus subtypes were seasonal H3N2 (57%), seasonal H1N1 (12%), and 2009 pandemic H1N1 (7%).

**Conclusions:**

The burden of influenza was small during 2007–2010 in this pediatric hospital in Kenya. Influenza A virus subtype H3N2 predominated, and 2009 pandemic influenza A virus subtype H1N1 had little impact.

A recent review of seasonal influenza during 1998–2009 revealed a paucity of epidemiological data throughout most of sub-Saharan Africa, including Kenya [1]. In particular, there were few influenza-associated data on age-specific incidence, mortality, seasonal variation, and relationship with common co-occurring conditions, including human immunodeficiency virus (HIV) infection, malnutrition, bacterial pneumonia, and malaria. In this study, we report the results of surveillance for influenza among patients presenting to a rural district hospital and outpatient clinics in coastal Kenya during 2007–2010. We used molecular diagnostic methods to distinguish influenza A, B, and C viruses and to subtype influenza A viruses. At admission to Kilifi District Hospital (KDH), detailed clinical and laboratory data were systematically collected, and residency within the surrounding Kilifi Health and Demographic Surveillance System (KHDSS) was ascertained. The study spans a period in which a new strain of influenza A virus, 2009 pandemic influenza A virus subtype H1N1 (A[H1N1]pdm09), spread worldwide and entered the study population, the occurrence of which was monitored through influenza virus subtyping.

## MATERIALS AND METHODS

### Study Population and Samples

We sampled children presenting to KDH and surrounding outpatient clinics located in Kilifi District on the coast of Kenya, approximately 60 km north of Mombasa. The district comprises a largely rural population of subsistence farmers and has an equatorial climate, with rain predominantly falling during April–July and October–December. KDH is the principal hospital facility for the population of the KHDSS. Further details of the study area and respiratory disease surveillance at KDH and local clinics can be found in previous reports (2–4, 13). Nasopharyngeal wash or aspirate specimens were collected from eligible children aged 1 day to 12 years from January 2007 through December 2010 and were either stored in viral transport medium at −80°C (2007–2009) prior to molecular screening or screened prior to freezing in raw form.

Inpatients were eligible if they were admitted to the hospital with cough or difficulty breathing and either lower-chest-wall indrawing (defined as severe pneumonia) or 1 or more of the following: cyanosis, prostration, unconsciousness, or an oxygen saturation level <90% (a modification of the World Health Organization criteria for very severe pneumonia). Children who were not residing in the KHDSS at admission, were admitted in extremis, were admitted for elective surgery, or received a diagnosis of neonatal tetanus were excluded. The following clinical and laboratory features obtained on admission or that relate to discharge outcome were compared between influenza-positive and influenza-negative children: duration of hospitalization >14 days, very severe pneumonia, wheezing, hypoxia (oxygen saturation level <90%, by fingertip pulse oximetry), circulatory shock (capillary refill time ≥3 seconds), severe anemia (hemoglobin level <5 g/dL), prematurity, congenital heart disease, positivity for HIV antibody (by 2 rapid tests), severe underweight (weight for age Z score ≤3), slide positivity for *Plasmodium* species, bacteremia, concurrent viral infection diagnosis, and death before discharge [2]. Outpatient recruits were a convenience sample of children aged <13 years, enrolled for broad comparison with hospital-admitted patients, who presented with either no signs of acute respiratory infection (non-ARI) or signs of upper respiratory tract infection (URTI) [2, 5]. Individuals with URTI had 1 or more of the following: cough, difficulty breathing, nasal discharge, runny or blocked nose, or sore throat. Written informed consent was obtained from the parent or guardian of subjects. This study was approved by the Kenyan National Ethical Review Committee and the University of Warwick Biomedical Research Ethics Subcommittee.

### Diagnostic Real-Time Reverse-Transcription Polymerase Chain Reaction (RT-PCR)

RNA was extracted from 140 µL of nasopharyngeal samples, using the Qiagen Viral RNA miniprep kit (Qiagen, United Kingdom), or from 200 µL of nasopharyngeal samples, using the total nucleic acid extraction kit (Roche Applied Science, Germany) with a MagNA Pure LC32 automated nucleic acid extractor, following the manufacturer's instructions. Diagnostic screening for viral targets was performed using real-time RT-PCR. For specimens from KDH inpatients in 2007 and from outpatients in 2007–2008, reactions were tested by multiplex real-time RT-PCR, using FRET hybridization probes as described by Lassaunière et al [6], with primers and probe targeting NS1 of influenza A virus and nucleoprotein of influenza B virus. All other samples were screened using the Taqman Qiagen Quantifast multiplex method on the ABI 7500 platform described by Hammitt et al, targeting the matrix protein for influenza A and C viruses and targeting NS for influenza B virus [3]. Concurrent targets in both RT-PCR assays included respiratory syncytial virus (RSV), adenovirus, rhinovirus, parainfluenza virus (PIV) 1–3, human metapneumovirus, coronavirus (CoV 229e, NL69, and OC43), and, differentially by assay, coronavirus Hong Kong [6] and PIV4 [3].

### Subtype Analysis

An aliquot of each of the influenza A virus–positive specimens was shipped to the National Influenza Center (NIC) in Nairobi for subtyping. Samples were subjected to RNA extraction using the QIAamp viral RNA isolation kit (Qiagen). Detection was performed using Invitrogen SuperScript III Platinum One-Step quantitative kit with primers and probes targeting seasonal influenza A virus H1N1 (A[H1N1]), A(H1N1)pdm09, and influenza A virus subtype H3N2 (A[H3N2]).

### Statistical Analysis

Statistical analysis was undertaken using Stata, version 11.0 (StataCorp, College Station, TX) and Microsoft Office Excel 2003 (Microsoft, Redmond, WA). The incidence of influenza among inpatients for age group *i*, *I(i)*, per 100 000 population per year was estimated on the basis of the equation *I(i)* = [C(i)/*N*(i)p(i)].100,100, where *C(i)* is the average number of children per year admitted who were resident in the KHDSS in age group *i*, *N(i)* the midsurvey KHDSS population in age group *i*, and *p(i)* is the proportion of eligible children tested for influenza (ie, we assumed that children who were not tested would have had the same prevalence of influenza as those who were tested and scaled the incidence accordingly). For pneumonia incidence estimates, *p(i)* is set to 1. The KDHSS population on 1 January 2009 was estimated to be 9451 individuals aged <1 year, 45 644 aged <5 years, and 108 708 aged <13 years. The population size and incidence estimation procedures have been described elsewhere [4]. The incidence estimation for the population proximal to the hospital was undertaken using cases involving children aged <5 years admitted from administrative sublocations within a 5-km radius of the hospital and the corresponding midpoint population estimate from the KHDSS (12 339 as of 1 January 2009). The Wilcoxon rank sum test was used to compare median ages; the χ^2^ or Fisher exact test was used to compare proportions, as appropriate; the Score test (procedure tabodds) was used to assess the trend in prevalence, by age; and the Poisson probability distribution was used to assess whether observed cases of influenza in specified quarters of the year exceeded the expected number of cases. Spearman rank correlation was used to test for a temporal association between monthly or quarterly numbers of influenza cases or influenza A virus infections and the number of cases of bacteremia or *Streptococcus pneumoniae* infection. The analysis was undertaken with cases of influenza and bacteremia temporally in phase or between 1 and 4 months time step out of phase. The association between positivity for any influenza type and laboratory or clinical features on admission was assessed using logistic regression, adjusted for age group, to obtain odds ratios (ORs) and 95% confidence intervals (95% CIs).

## RESULTS

Over the 4-year period, there were 2429 admissions to KDH involving individuals who were eligible for the study (57% were boys; median age, 9 months [interquartile range {IQR}, 3–22 months]). A total of 503 (21%) had very severe pneumonia (50% were boys; median age, 10 months [IQR, 2–34 months]; 55% were infants), and the in-hospital case-fatality rate was 6.5%. Of the eligible inpatients, 2002 (82%) were tested for influenza (57% were boys; median age, 9 months [IQR, 3–21 months]; 58% were infants), and this percentage did not differ among those aged <1 year, 1–4 years, and ≥5 years (*P* = .333); 387 (19%) had very severe pneumonia (52% were boys; median age, 10 months [IQR, 2–34 months]; 53% were infants). Stratified by severity, 84% of eligible inpatients with severe pneumonia were tested, compared with 77% of eligible inpatients with very severe pneumonia (*P* = .001). The case-fatality rate among inpatients who were untested was significantly higher than among those who were tested (69 of 427 [16%] vs 88 of 2002 [4.4%]; *P* < .0001). Reasons for not testing comprised consent refusal (63%), early discharge (26%), and death (11%) before sampling.

Within the same period, 527 outpatients were recruited and tested for influenza from May 2007 through March 2008 (57 with non-ARI and 96 with URTI) and again from March 2010 through December 2010 (139 with non-ARI and 235 with URTI). Cumulative non-ARI cases numbered 15, 40, 76 and 65, for quarters 1–4, respectively, and cumulative URTI cases numbered 47, 95, 99, and 90, respectively. Overall, 196 outpatients had non-ARI (median age, 13 months [IQR, 5–26 months]; 47% were infants), and 331 had URTI (median age, 19 months [IQR, 8–39 months]; 37% were infants). Compared with the median age of inpatients, the median ages of outpatients with non-ARI (*P* = .004) or URTI (*P* ≤ .0001) were higher.

### Prevalence, Disease Association, and Incidence

The prevalence of influenza virus of any type was 4.9% (99 of 2002 cases) among inpatients; 4.7% (76 of 1615) had severe pneumonia, and 5.9% (23 of 387) had very severe pneumonia (*P* = .299). Among outpatients, the prevalence of influenza virus of any type was 3.9% (13 of 331) among those with URTI and 0.5% (1 of 196) among those with non-ARI. Data stratified by virus type are presented in Table 1. Influenza A virus was the most prevalent type among outpatients with pneumonia (3.5%) and outpatients with URTI (3.3%). These proportions were unaltered by restricting the analysis to children <5 years of age. Among outpatients classified as having non-ARI, there were no cases of influenza B or C virus infection; influenza A virus was detected in 1 child.

**Table 1.** Distribution of Influenza Viruses Diagnosed by Molecular Methods Among Children, by Presenting Condition, From Kilifi, Kenya, 2007–2010

Condition	No. of Children		Influenza Virus Detected, by Type, No. (%^b^) of Children			
	Eligible	Tested	Any^a^	A	B	C^c^
NARI	196	196	1 (0.5)	1 (0.5)	0	0
URTI	331	331	13 (3.9)	11 (3.3)	1 (0.3)	2 (0.9)
Severe pneumonia	1926	1615	76 (4.7)	57 (3.5)	13 (0.8)	7 (0.6)
Very severe pneumonia	503	387	23 (5.9)	14 (3.6)	6 (1.6)	4 (1.4)
All pneumonia (severe and very severe)	2429	2002	99 (4.9)	71 (3.5)	19 (0.9)	11 (0.8)
Total	2956	2529	113 (4.5)	83 (3.3)	20 (0.8)	13 (0.7)

All children resided in the Kilifi Health and Demographic Surveillance System.

Abbreviations: NARI, no acute respiratory infection; URTI, upper respiratory tract infection.

^a^ Includes influenza A, B, or C viruses. There were 13 influenza diagnoses among outpatients with URTI, but 14 influenza viruses were detected because 1 child was coinfected (with influenza A and C viruses), and there were 99 influenza diagnoses among all inpatients with pneumonia, but 101 influenza viruses were detected because 2 children were coinfected (with influenza A and B viruses in one and influenza A and C viruses in the other).

^b^ % is number detected / number tested

^c^ Because samples collected during 2007 were not tested for influenza C virus, percentages are based on the following denominators: NARI, 139; URTI, 235; severe pneumonia, 1126; and very severe pneumonia, 288.

The estimated incidence of severe or very severe pneumonia among children <1 year of age, <5 years of age, and <13 years of age was 3902 (95% CI, 3708–4106), 1321 (95% CI, 1269–1375), and 589 (95% CI, 567–612) cases per 100 000 population per year, respectively. Correspondingly, for all inpatients with influenza, the incidence of severe or very severe pneumonia for those aged <1 year, <5 years, and <13 years was 154 (95% CI, 116–204), 60 (95% CI, 49–74), and 28 (95% CI, 23–34) cases per 100 000 per year, respectively, after scaling for the proportion of patients who were tested for pneumonia (ie, 0.82). For inpatients with influenza A virus infection, the incidence of severe or very severe pneumonia among children aged <1 year, <5 years, and <13 years, was 106 (95% CI, 75–149), 43 (95% CI, 34–55), and 20 (95% CI, 16–25) cases per 100 000 per year, respectively. Considering only inpatients <5 years of age who were admitted from locations within a 5-km radius of KDH, the incidence estimates (using a scaling factor, *p*, of 0.78) of severe or very severe pneumonia, influenza, and influenza A virus infection were 1815 (95% CI, 1700–1938), 106 (95% CI, 78–144), and 70 (95% CI, 48–102) cases per 100 000 per year, respectively. Comparative estimates of incidence by age group for other virus groups are presented in Supplementary Table 2. Rhinovirus and RSV infection had higher incidences than that for influenza virus by factors of 4–6 and 5–8, respectively, according to age group.

### Distribution of Influenza Virus Infection, by Season and by Age

**Figure 1.**
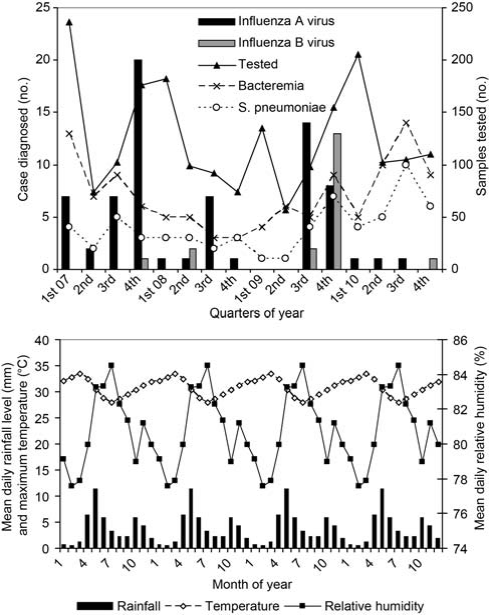
Temporal distribution, by quarter, of molecular diagnoses of influenza A virus infection (dark bars) and influenza B virus infection (light bars) in nasopharyngeal samples collected from children with severe or very severe pneumonia who were admitted to Kilifi District Hospital, 2007–2010. Also, on the same axis are shown the quarterly number of admissions with bacteremia (x markers) and *S. pneumoniae* (o markers). The numbers of nasopharyngeal swab specimens collected each quarter are shown on the secondary Y axis (triangle markers). *B,* Monthly weather patterns averaged during 2007–2010.

The seasonal patterns of influenza A and B virus infection are shown in Figure 1. Over the 4-year period, influenza A and B virus infection showed a significantly higher occurrence among inpatients during quarters 3 and 4, relative to the average for all quarters (22.5 cases expected vs 37.5 cases observed; *P* = .003). There was very little influenza activity in 2010. The majority of influenza B virus infections occurred in the fourth quarter of 2009. Most influenza cases occurred after the main period of rainfall (April–July) and before peak temperatures (first quarter; Figure 1). Cases of bacteremia, and specifically *S. pneumoniae* infection, by quarter, are shown in Figure 1. No statistically significant correlation between influenza cases (or influenza A virus infections) and occurrence of bacteremia (or *S. pneumoniae* infection) was identified, either concurrently or delayed (*P* > .05).

**Figure 2.**
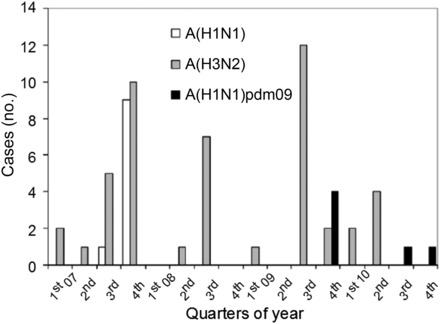
Temporal distribution, by quarter, of influenza A virus subtypes for outpatients and inpatients combined from Kilifi District, coastal Kenya, 2007–2010.

Of the 70 of 83 influenza A virus–positive samples sent to the NIC for subtyping, 42 (60%) were A(H3N2) (median age, 13 months [range, 0–134 months]), 10 (14%) were A(H1N1) (median age, 21 months [range, 4–127 months]), and 4 (6%) were A(H1N1)pdm09 (median age, 31 months [range, 17–43 months]), with 14 failing to subtype (7 were unconfirmed as influenza A virus). Among outpatients aged <13 years who presented with URTI, 7 had A(H3N2), 3 had A(H1N1), and 0 had A(H1N1)pdm09. The distribution of subtypes over time is shown in Figure 2. A(H3N2) circulated in all years, and A(H1N1) was confined to 2007; A(H1N1)pdm09 occurred in late 2009 and again in late 2010.

The age distributions of diagnoses of influenza A, B, or C virus infection over the 4 years of surveillance are shown in Supplementary Figure 1 and follow a similar pattern (Supplementary Figure 1*A*), with around 50% of cases in infants (46% had influenza A virus infection, 47% had influenza B virus infection, and 66% had influenza C virus infection; *P* = .632). The proportion of inpatients with pneumonia who were found to be influenza positive showed a trend for increase with increasing age (Supplementary Figure 1*B*). In the case of influenza A virus infection, the trend was significant: 1.7% of children aged 0–2 months had influenza A virus infection, compared with >5% of children aged ≥24 months (*P* = .005).

### Clinical Characteristics and Disease Severity

Analysis of the association between a diagnosis of infection with any influenza virus and components of a set of severity features yielded an increased odds of hypoxia among children with influenza, compared with those without influenza (age-adjusted OR, 1.78; 95% CI, 1.04–1.96). No other severity feature was significantly associated with influenza (Supplementary Table 1). There were 4 deaths (age range, 7–32 months) among the 99 inpatients with influenza; one was infected with influenza A virus, 2 were infected with influenza B virus, and 1 was infected with influenza C virus. Three were positive for HIV antibody, severely malnourished (weight-for-age Z score, ≤4), and had a discharge diagnosis including immunosuppression, and 1 inpatient (who was infected with influenza A virus) had chronic heart disease.

## DISCUSSION

Our study identified all 3 influenza viruses in circulation in this rural coastal Kenya location among patients hospitalized with severe or very severe pneumonia and among outpatients with URTI. However, the prevalence of infection with any influenza virus was low among inpatients with severe or very severe pneumonia (4.9%) and among outpatients with URTI (3.9%). Influenza A virus predominated, with identification in 3.5% of inpatients and 3.3% of outpatients with URTI. Correspondingly, relative to the incidence of severe or very severe pneumonia among hospitalized children aged <5 years (1323 cases per 100 000 per year), the incidences of influenza (64 cases per 100 000 per year) and influenza A virus infection (46 cases per 100 000 per year) were low. There was near absence of influenza in the convenience sample of children without signs of respiratory illness. Our inpatient data are consistent with the results of a recent review of data on seasonal influenza from 15 published studies in sub-Saharan Africa [1], which reported a median prevalence of 6.0% among hospitalized pediatric patients, with a range of 0%–16%. In the same review, the median prevalence of influenza among outpatients with ARI was higher than we found, at 10% (range, 1%–25%; 11 studies), but comparisons should be cautioned because of a number of methodological differences. Exploration of a range of severity features and concurrent illnesses revealed hypoxia to be more commonly associated with influenza among hospitalized children.

Calculation of the incidence of influenza-associated severe disease on the basis of hospital admission data is likely to underestimate the true burden in the community, as a result of the relationship between healthcare access and distance from the hospital. This is supported in the analysis, where it was shown that the incidence of influenza-associated admissions among children with severe or very severe pneumonia was about 70% greater in the population proximal to the hospital. We have previously shown a similar distance decay for severe rotavirus diarrhea [7] and severe RSV-associated pneumonia [4] and pneumonia and meningitis [8]. Furthermore, a previous study of RSV among infants and young children in the HDSS revealed that roughly 4 in 5 children identified with severe pneumonia in the outpatient setting were not admitted to the local hospital [9].

Notwithstanding this underestimation, it is clear that the incidence of influenza-associated hospital admissions is significantly lower than that associated with either rhinovirus or RSV infection. While the etiology of rhinovirus as the causative agent of lower respiratory tract disease may be in question [10], this is not the case for RSV, which is known to be a major cause of infant and childhood lower bronchiolitis and pneumonia in sub-Saharan Africa and globally [11]. While RSV is invariably among the most prevalent viruses in children admitted with lower respiratory tract illness, it is not always dominant over influenza [1]. In Kenya, contemporary data on the relative prevalence of respiratory viruses among pneumonia-related admissions to the hospital are largely absent. Further data are clearly needed in Kenya to gauge the relative burden of disease due to respiratory viruses and thereby help support future health policy planning.

In terms of seasonality, there was increased occurrence in the third and fourth quarters of each year, most notably for influenza A virus infection, except in 2010. These periods are characteristically times of lower rainfall levels (referred to locally as “second rains”), intermediate temperatures, and relative lower humidity. During the study period, A(H1N1)pdm09 entered Kenya [12], and cases of A(H1N1)pdm09 infection were identified in KDH from late 2010. It is possible that the introduction of A(H1N1)pdm09 disrupted the normal pattern of A(H3N2) activity in 2010; only A(H1N1)pdm09 was observed in the latter quarters of 2010. Continued surveillance will reveal whether A(H1N1)pdm09 has any long-term effect on the circulation patterns of other influenza subtypes. However, in general, the contribution of A(H1N1)pdm09 to the burden of hospitalization-associated pneumonia was minimal in this setting. The possibility exists that the burden of influenza was underestimated because of associated, but delayed, invasive bacterial disease. However, we identified no evidence for an increased number of admissions in which bacteria (or *S. pneumoniae,* in particular) were detected in blood cultures during the quarter following peak occurrences of influenza.

During the A(H1N1)pdm09 infection pandemic, antiviral therapy (oseltamivir) was prescribed to children admitted to KDH with severe acute respiratory illness on a presumptive basis (ie, prior to laboratory confirmation of influenza.) This would not have altered the pattern of observation of influenza described in this study, because nasopharyngeal specimens were collected prior to treatment with the antiviral. Within the surrounding community, A(H1N1)pdm09 vaccination was undertaken in 2010 but was limited to target groups, including healthcare workers, pregnant women, and patients with chronic disease, totaling 2203 subjects (Kenya Ministry of Health, personal communication). This number and the age group of subjects receiving vaccine would not have altered the pattern of A(H1N1) infection occurrence described in this study.

Half of the influenza cases occurred in infants, and the proportion of cases rapidly declined with age into older age groups. However, we noted that within the age group that we studied, the prevalence of influenza increased with age, suggesting that relative to other causes of cases of severe or very severe pneumonia associated with admission, those caused by influenza decline less rapidly with age.

A limitation of the present study is the failure to recruit and test samples from approximately 20% of eligible children. The reasons for this have been previously described [2, 4] and were primarily due to parental refusal and, to a lesser extent, to discharge or death before sampling. Failure to recruit and test was more common for critically ill children and for those who died while in the hospital. This almost certainly led to an underestimation of severity associated with influenza. A further limitation of the study is that results are based only on nasopharyngeal samples, and we now have definitive evidence that an oropharyngeal swab specimen provides added diagnostic value for detecting influenza in our setting (22% increased detection; 95% CI, 9–42), compared with nasopharyngeal specimens alone [3]. The low prevalence of influenza viruses in this study limits the power of the analysis to identify associations between virus presence and specific clinical features or coinfections. Our study involved sampling of 2000 children; a definitive investigation of clinical associations will require a considerably larger sample size or a location with a markedly higher incidence of influenza A virus infection. We report very low prevalence of influenza in outpatient children without signs of acute respiratory infection, suggesting that influenza virus is rarely the cause of asymptomatic infection, and the data also suggest influenza is the cause of only 4% of URTI cases. Further interpretation of these data with regard to the association between influenza and severe disease is unwarranted since outpatient sampling was not contemporaneous throughout the period of surveillance of hospitalized patients, and collection was not frequency matched by age and location within the KHDSS. We therefore await the complete results of a larger and better-designed case-control study, which is ongoing.

In conclusion, although the incidence of influenza was underestimated in this study, it is clear that influenza contributes only a small proportion of the total burden of hospitalization-associated severe and very severe pneumonia among children in this rural coastal Kenya setting. Influenza A virus is the dominant influenza virus causing pediatric severe and very severe pneumonia. A seasonal signature for influenza was evident, but no temporal association was identified with invasive bacterial disease. Although A(H1N1)pdm09 infection was observed, its contribution to disease was not substantial. Hypoxia was more frequently identified among patients with influenza, and immunosuppression, severe malnutrition, or chronic heart disease were identified in all of the 4 influenza-associated deaths. Given the low influenza prevalence, larger studies are required to investigate associations between influenza and disease severity or prevalent conditions, such as malaria, HIV infection, or malnutrition. Additional comparative studies on viral diagnoses in severe pneumonia hospital admissions are warranted elsewhere in Kenya. Such data may be informative to the Kenya Ministry of Health in their assessment of the role for influenza antivirals and vaccination in Kenya.

## Supplementary Data

Supplementary materials are available at *The Journal of Infectious Diseases* online (http://jid.oxfordjournals.org/). Supplementary materials consist of data provided by the author that are published to benefit the reader. The posted materials are not copyedited. The contents of all supplementary data are the sole responsibility of the authors. Questions or messages regarding errors should be addressed to the author.
